# Early Versus Late Enzyme Replacement Therapy in Siblings With Morquio A Syndrome: Insights Into Therapeutic Timing

**DOI:** 10.1002/jmd2.70095

**Published:** 2026-07-26

**Authors:** Shinjie Choi, Hyoungmin Kim, Tae‐Joon Cho, Jung Min Ko

**Affiliations:** ^1^ Department of Pediatrics Seoul National University College of Medicine Seoul Korea; ^2^ Department of Orthopaedic Surgery Seoul National University College of Medicine Seoul Korea; ^3^ Department of Pediatric Orthopaedics Seoul National University Children's Hospital Seoul Korea; ^4^ Rare Disease Center Seoul National University Hospital Seoul Korea

**Keywords:** enzyme replacement therapy, kyphosis, mucopolysaccharidoses, spinal stenosis

## Abstract

Enzyme replacement therapy (ERT) with elosulfase alfa is the only approved treatment for mucopolysaccharidosis type IVA. This case report delineates the 5‐year outcomes of ERT in two Korean siblings with mucopolysaccharidosis type IVA, with the younger sibling initiating treatment at 0.8 years of age and the older one at 5.4 years. Both patients exhibited a progressive decline in their height standard deviation scores, with trajectories paralleling the natural history curves of the disease. At 5.2 years of age, persistent skeletal dysplasia was evident in both siblings. However, the younger sibling demonstrated attenuated disease severity, lacking cervical myelopathy or spinal stenosis requiring C1 laminoplasty. Cardiorespiratory assessment revealed normalized left ventricular mass index z‐scores, stable ejection fractions, and the absence of valvular pathology. Overall, these findings suggest that early ERT attenuates severe spinal and upper body manifestations but does not prevent lower limb skeletal progression, highlighting the need for early therapeutic initiation along with orthopedic intervention to preserve cardiorespiratory parameters and functional independence.

## Summary

Observations from this sibling pair suggest that early infancy may represent a critical therapeutic window to mitigate severe cervical complications while also acknowledging the limited skeletal response to intravenous ERT due to the avascular nature of cartilage.

## 1 Introduction

Mucopolysaccharidosis type IVA (MPS IVA; OMIM #253000), also known as Morquio A syndrome, is a rare autosomal recessive lysosomal storage disorder caused by a deficiency of *N*‐acetylgalactosamine‐6‐sulfatase (GALNS), which is encoded by *GALNS*, leading to defective lysosomal degradation of keratan sulfate (KS) and chondroitin‐6‐sulfate [1, 2, 3]. A definitive diagnosis requires confirmation of decreased GALNS activity in leukocytes or fibroblasts or identification of biallelic pathogenic variants of *GALNS* [1].

MPS IVA is a heterogeneous progressive disorder predominantly characterized by skeletal and joint abnormalities, including genu valgum, hip subluxation or dysplasia, spinal cord compression, and thoracolumbar kyphoscoliosis. Additional clinical features include corneal clouding and hearing impairment [1, 2, 3].

Elosulfase alfa, approved for Korean National Health Insurance coverage in 2016, is currently the only licensed enzyme replacement therapy (ERT). ERT significantly improves the 6‐min walk test (6MWT) performance and respiratory function. However, its efficacy in the avascular cartilage and growth plates remains suboptimal owing to limited tissue penetration [1, 2, 3, 4].

This case report describes the 5‐year clinical outcomes of ERT in two Korean siblings with MPS IVA who initiated therapy at different ages, highlighting how early treatment initiation may modify disease progression.

## 2 Methods

### 2.1 Patient Information

This report presents the clinical course of two Korean siblings diagnosed with MPS IVA who received ERT over a 5‐year period from September 2020 to November 2025. The patients were born to non‐consanguineous Korean parents. Height was measured using a Harpenden stadiometer (Holtain, Crymych, Wales, UK), with standard deviation scores calculated relative to the 2017 Korean National Growth Charts (SDS_K) [5] and the growth reference for untreated MPS IVA (SDS_M) [6, 7]. Longitudinal growth outcomes were evaluated by assessing the height trajectories relative to both healthy pediatric norms and the natural history of the disease.

### 2.2 Biochemical and Genetic Analyses

GALNS activity in peripheral blood leukocytes was quantified using fluorometric assays [8]. Informed parental consent was obtained for the molecular genetic testing. Urinary glycosaminoglycans were qualitatively and quantitatively analyzed using liquid chromatography–tandem mass spectrometry [9]. Genomic DNA was extracted from peripheral blood leukocytes, and targeted mucopolysaccharidosis panel sequencing confirmed compound heterozygous *GALNS* variants in Sibling 1. Family screening for the identified variants using Sanger sequencing confirmed that the variants were *trans*, and Sibling 2 was diagnosed with MPS IVA.

### 2.3 Assessments During ERT

Patients received weekly elosulfase alfa (Vimizim, 2.0 mg/kg) infusions. Pre‐ and post‐ERT skeletal evaluations by an orthopedic specialist included annual plain radiographs of the hips, knees, ankles, and spine. Cervical spine magnetic resonance imaging was performed at baseline and repeated based on myelopathy symptoms or signs. Echocardiographic assessments, including heart valve or aortic root abnormalities, ejection fraction, and left ventricular mass index (LVMI), were conducted biannually [10]. LVMI z‐scores were calculated according to reference equations from the Pediatric Heart Network Normal Echocardiogram Database using the body surface area derived from the Haycock formula [11]. Pulmonary function tests, including forced vital capacity and expiratory volume in 1 s, were performed in Sibling 1 after 6 years of age [12]. Endurance was quantified using the 6MWT in ambulatory patients [13]. Functional independence was assessed using the Pediatric Functional Independence Measure (FIM), which comprises 18 items across self‐care, mobility, and cognition (126 points) [14]. Anthropometric measurements, echocardiography, pulmonary function tests, the 6MWT, and FIM were assessed annually. Ophthalmic and otorhinolaryngological examinations including age‐appropriate pure‐tone audiometry and auditory brainstem responses were conducted at baseline and annually thereafter.

### 2.4 Clinical Outcomes and Visualization

Due to the small sample size inherent to a two‐patient sibling case study, clinical and radiographic outcomes were evaluated strictly descriptively. A cross‐sectional comparison of clinical outcomes was conducted at an age‐matched time point (5.2 years). To observe the clinical changes over time, baseline and the most recent follow‐up values were reported. All data visualization, including the generation of clinical growth trajectories, was performed using R version 4.5.1 (R Foundation for Statistical Computing, Vienna, Austria).

## 3 Results

### 3.1 Case Presentation

Sibling 1 (the older brother) was born at 39 weeks of gestation (birth weight, 3.83 kg; SDS_K, 0.9). He was the first child of healthy non‐consanguineous parents. The patient achieved independent ambulation at 12 months of age. Progressive scoliosis was noted at 1 year of age, with mild corneal clouding observed at 5 years of age. Orthopedic evaluation at 5 years of age prompted GALNS activity and targeted gene panel analyses, which confirmed MPS IVA. Weekly ERT was initiated at 5.4 years, followed by French‐door laminoplasty extending to C1 for compressive myelopathy. No postoperative neurological deterioration has been reported to date.

Sibling 2 (the younger sister) was born at 38 weeks of gestation (birth weight, 3.73 kg; SDS_K, 1.0). She was diagnosed with MPS IVA at 7 months of age via family screening prompted by the diagnosis of Sibling 1, and ERT was initiated at 9 months of age.

### 3.2 Biochemical and Genetic Diagnosis

GALNS activity was markedly reduced in both siblings. They inherited the *GALNS* variants c.752G>A (p.R251Q; likely pathogenic) and c.1426C>T (p.Q476*; pathogenic) from their fathers and mothers, respectively, confirming compound heterozygosity in the *trans* configuration.

### 3.3 Growth and Skeletal Outcomes

**FIGURE 1:**
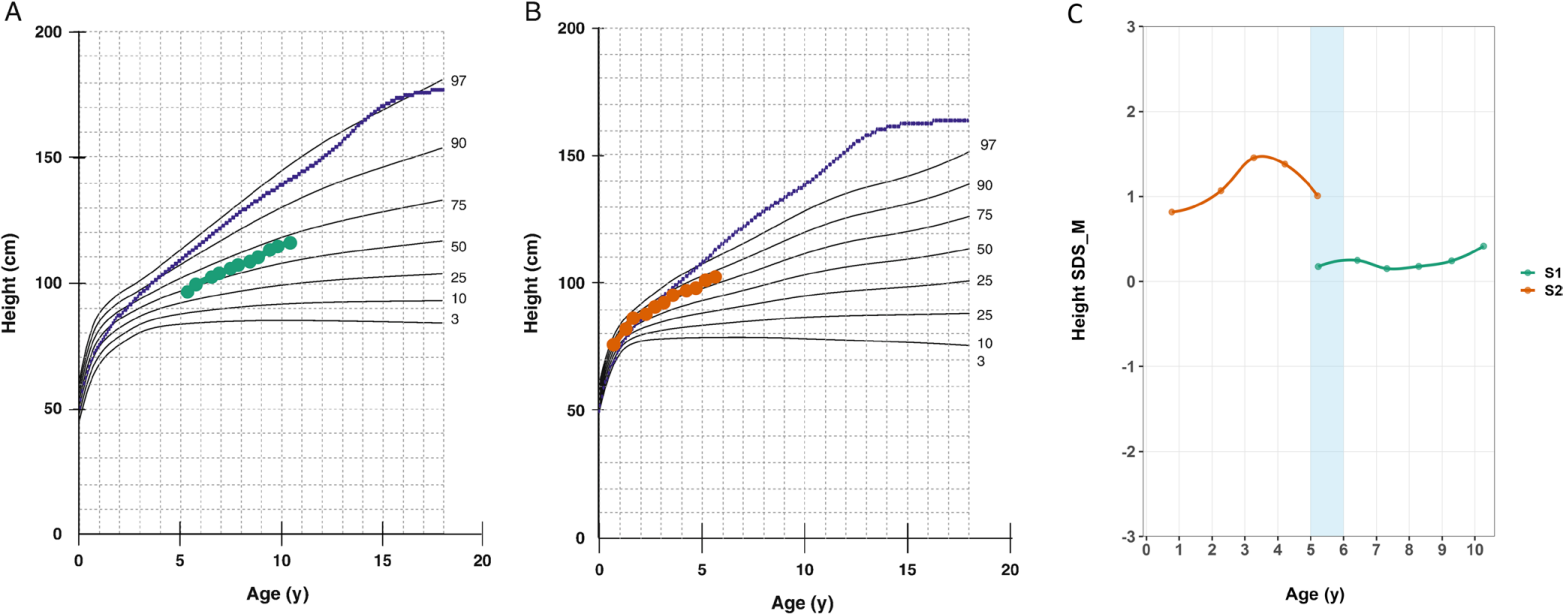
Height trajectories of siblings with mucopolysaccharidosis IVA (MPS IVA) on MPS IVA‐specific growth charts. (A) Sibling 1 (green). (B) Sibling 2 (orange). Purple dotted line: 50th percentile for healthy children (Centers for Disease Control and Prevention growth charts). (C) Height standard deviation score curves for siblings with MPS IVA, based on the growth reference for untreated MPS IVA (SDS_M).

**FIGURE 2:**
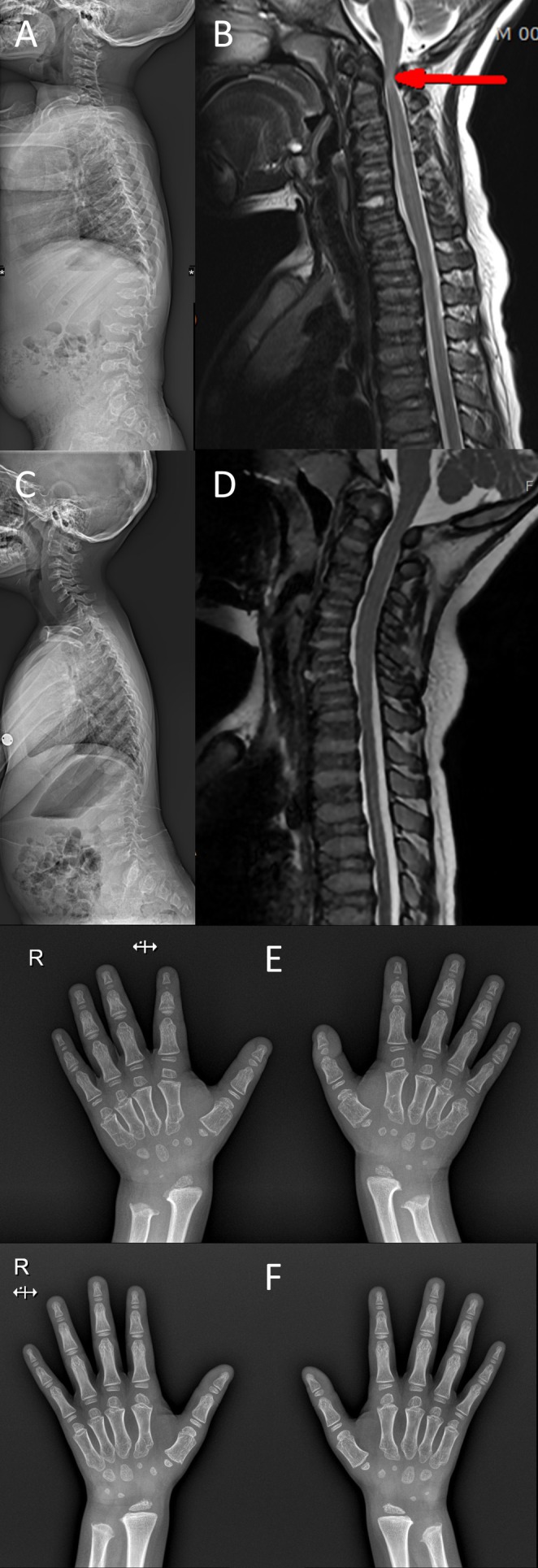
Radiographs and spinal magnetic resonance imaging (MRI) of siblings with mucopolysaccharidosis IVA. (A) Spinal column radiograph (Sibling 1, 5.4 years). (B) Spinal MRI (Sibling 1, 5.2 years) showing spinal cord compression at the cervicomedullary junction (C1 level). (C) Spinal column radiograph (Sibling 2, 5.2 years). (D) Spinal MRI (Sibling 2, 4.7 years) showing no spinal cord compression. (E) Hand radiograph (Sibling 1, 5.0 years). (F) Hand radiograph (Sibling 2, 4.2 years).

Both siblings exhibited persistent linear growth deficits. Compared with SDS_M, the trajectories paralleled the natural history curves (Figure 1). At 5.2 years, both siblings had kyphosis and acetabular dysplasia, with genu valgum. However, Sibling 1 presented with C1‐level cervical myelopathy and cervical canal stenosis (Table 1; Figure 2) and required orthopedic surgery at 5.4 years of age, shortly after diagnosis. The extent of the spinal and lower‐extremity skeletal deformities for both patients was further characterized using quantitative orthopedic and radiographic measurements, as detailed in Table 1.

**TABLE 1:** Characteristics of siblings with mucopolysaccharidosis type IVA: Cross‐sectional comparison at 5.2 years (pre‐enzyme replacement therapy [ERT], Sibling 1) versus 4.2 years (ERT, Sibling 2) and longitudinal clinical outcomes of ERT over 5 years.

Cross‐sectional comparison at age 5.2 years		
Sex	Sibling 1 (pre‐ERT)	Sibling 2 (4.2‐years ERT)
	M	F
Height SDS_K	−2.9	−2.1
Height SDS_M	0.2	1.0
Skeletal symptoms	Kyphosis, acetabular dysplasia of the hips, cervical myelopathy, cervical stenosis, pectus carinatum, ulnar deviation of the wrist, and genu valgum	Kyphosis, acetabular dysplasia of the hips, and genu valgum
Non‐skeletal symptoms	Corneal clouding, middle ear effusion, and recurrent acute otitis media	—
6MWT distances (m)	205	260
FIM score	81	110
EF (%)	71.0	75.9
LVMI z‐score	2.8	−0.2
Cervical canal antero‐posterior diameter, C1 level (mm)	6.0	9.0
Reimers' migration percentage (%)	38.7	17.6
Angle of inclination of the hip (°)	133.8	131.7
Acetabular index (°)	26.0	31.1
Mechanical tibiofemoral angle (°)	3.3	7.2
Lateral distal femoral angle (°)	89.5	88.8
Medial proximal tibial angle (°)	96.7	95.0

Longitudinal clinical outcomes of ERT over 5 years				
	Sibling 1		Sibling 2	
	Baseline	Latest	Baseline	Latest
Height SDS_K	−2.9	−4.7	2.6	−2.6
Height SDS_M	0.2	0.4	0.8	1.0
6MWT distances (m)	205	225	172.2	278
FIM score	81	120	26	111
EF (%)	71.0	77.1	68.3	68.8
LVMI z‐score	2.8	−0.02	3.9	0.1
Urinary KS (μg/mL)	111.1	59.2	6.2	45.4

Abbreviations: 6MWT, 6‐min walk test; EF, ejection fraction; ERT, enzyme replacement therapy; FIM, functional independence measure; KS, keratan sulfate; LVMI, left ventricular mass index; SDS_K, standard deviation score based on the Korean National Growth Charts; SDS_M, standard deviation score based on the growth reference for untreated MPS IVA.

### 3.4 Cardiorespiratory and Functional Outcomes

The LVMI z‐scores decreased progressively in both siblings during ERT. In Sibling 1, forced vital capacity and expiratory volume in 1 s remained within normal limits but showed a downward trajectory. Moreover, the 6MWT distance demonstrated initial gains after ERT initiation, followed by a decline in both siblings. The FIM scores increased steadily throughout ERT in both siblings (Table 1). Importantly, when comparing the functional trajectories at age‐matched time points, Sibling 2 consistently achieved higher 6MWT distances and FIM scores than Sibling 1.

### 3.5 Ophthalmic and Otorhinolaryngologic Findings

At ERT initiation, Sibling 1 exhibited mild bilateral corneal clouding, which had not progressed for more than 5 years. Pre‐ERT recurrent acute otitis media affected Sibling 1; however, audiometry revealed no conductive hearing loss. In contrast, Sibling 2 showed normal findings.

## 4 Discussion

The results demonstrate a sustained response to elosulfase alfa, with improvements in cardiorespiratory parameters and functional independence, despite limited growth gains and persistent skeletal dysplasia on imaging in both siblings with MPS IVA.

Both siblings exhibited a progressive decline in height standard deviation scores relative to healthy Korean norms (SDS_K). Furthermore, their growth trajectories closely paralleled the disease‐specific reference curves for untreated patients (SDS_M), suggesting that even with early ERT initiation in Sibling 2, the natural history of linear growth was not significantly modified. This lack of deviation from untreated growth trajectories supports the limited effect of intravenous elosulfase alfa on longitudinal bone growth, likely reflecting restricted penetration into the avascular growth plates [4]. This pattern is consistent with previous reports of attenuated growth in MPS IVA due to underlying skeletal dysplasia [15, 16, 17, 18].

Sibling 1 showed extensive skeletal involvement requiring C1 laminoplasty at 5.4 years, whereas Sibling 2 exhibited milder features. Early ERT may have attenuated severe spinal progression in Sibling 2, despite shared lower limb deformities. This divergent outcome closely mirrors the findings of Barak et al. [17], who reported that initiating ERT at 11 months of age in a younger sibling preserved stability of the cervical spine compared to an older sibling treated at 54 months, yet failed to prevent the progression of severe orthopedic deformities. Consistent with this previous report, our observations suggest that the first year of life may represent a critical therapeutic window to mitigate severe cervical myelopathy and upper body manifestations. However, this pattern also reflects a well‐recognized physiological limitation of ERT, namely its restricted access to avascular cartilage and growth plates. Intravenously administered elosulfase alfa is rapidly cleared from systemic circulation, with a half‐life of approximately 40 min, due to preferential receptor‐mediated uptake by visceral organs via mannose‐6‐phosphate and mannose receptors. As a result, enzyme delivery to chondrocytes within the growth plates is likely limited. Consequently, even with prolonged administration, conventional ERT has a limited capacity to modify growth plate pathology and skeletal dysplasia in MPS IVA [4]. Given the lack of clinical evidence demonstrating effective delivery of intravenously administered enzyme to avascular tissues, early treatment is unlikely to prevent lower limb skeletal manifestations. Therefore, findings from sibling studies must be interpreted cautiously on a case‐by‐case basis, highlighting the importance of proactive orthopedic management rather than broad generalization.

Previous studies of MPS VI siblings have shown conflicting early ERT skeletal outcomes [19, 20], suggesting differences in subtype‐specific glycosaminoglycan storage and responsiveness. In MPS IVA, KS and chondroitin‐6‐sulfate accumulation in the avascular cartilage and growth plates limit intravenous ERT access, thereby explaining the attenuated skeletal response compared with other subtypes [4].

LVMI z‐scores normalized progressively in both siblings, suggesting the cardioprotective role of ERT irrespective of initiation age. Stable ejection fractions and the absence of valvular abnormalities further supported these findings, which are consistent with previous reports [18, 21]. Baseline pulmonary function tests in Sibling 1 remained within normal limits but showed a mild downward trend due to skeletal involvement, consistent with a previous report [12].

The potential protective effect of early intervention on functional capacity is suggested by the cross‐sectional comparison of the siblings. Sibling 2 consistently achieved higher 6MWT distances and FIM scores than Sibling 1 at age‐matched time points. Although the 6MWT and FIM trajectories corroborate the functional improvements reported in previous studies [13, 22, 23, 24], subnormal final 6MWT distances relative to healthy pediatric norms suggest residual activity limitations. These findings should be interpreted with caution, particularly in Sibling 2, who initiated therapy at 0.8 years of age. In such a young child, functional gains are likely influenced by normative neurodevelopmental maturation and gross motor acquisition, making it difficult to distinguish therapeutic effects from natural developmental progress [10, 13, 25]. Furthermore, although the siblings share an identical compound heterozygous *GALNS* genotype, they are of different sexes. We cannot exclude the possibility that sex‐related differences in endocrine function, bone metabolism, or prepubertal growth velocity may have contributed to the observed variations and therapeutic responsiveness.

Given the complexities, our findings highlight a distinct contrast between soft tissue and skeletal outcomes. To clarify this distinction, organ systems that appear responsive versus non‐responsive to early ERT are summarized alongside existing literature in Table 2.

**TABLE 2:** Organ‐specific responsiveness to early enzyme replacement therapy in patients with mucopolysaccharidosis type IVA: Current findings and supporting literature.

Organ system	Therapeutic responsiveness	Clinical outcomes	Supporting literature
Cardiovascular	Responsive	Normalization of left ventricular mass index z‐scores; maintenance of stable ejection fraction and absence of valvular pathology.	Frigeni et al. [18]
Endurance and functional independence	Responsive	Improvements in 6‐min walk test distances and functional independence measure scores.	Barak et al. [17] Ficicioglu et al. [23] Burton et al. [24]
Cervical myelopathy	Responsive	Prevention or delay of compressive cervical myelopathy requiring surgical intervention.	Barak et al. [17]
Linear growth	Non‐responsive	Persistent linear growth deficits, but paralleled untreated disease‐specific reference curves.	Do Cao et al. [15] Doherty et al. [16] Frigeni et al. [18]
Lower limb skeletal	Non‐responsive	Progression of acetabular dysplasia, genu valgum, and bone deformity.	Barak et al. [17] Frigeni et al. [18]

As a case report of two siblings, this study has some limitations, including a small sample size and the absence of a control group, which affect the generalizability of the findings. Moreover, the assessments reflect routine clinical observations rather than controlled trial data, and the 5‐year follow‐up may not capture lifelong outcomes. As this was a retrospective clinical study, therapeutic monitoring was limited to urinary KS, a recognized pharmacodynamic marker. Blood KS, which may serve as a more robust surrogate biomarker for skeletal outcomes, was not assessed and represents a limitation of this study [4, 26]. In addition, analyses were restricted to diagnostic genetic sequencing and leukocyte enzyme assays, and no in vitro functional studies were performed to evaluate residual GALNS activity. Importantly, the divergent skeletal outcomes observed in this single sibling pair should not be broadly generalized to all early‐treated cases.

In conclusion, ERT was associated with cardiorespiratory and functional gains in the two siblings with MPS IVA despite persistent growth deficits and progressive skeletal dysplasia. Although early ERT initiation appeared to attenuate severe cervical manifestations, its failure to prevent lower limb involvement highlights the importance of concurrent orthopedic management. While clinical outcomes must be evaluated individually, the trajectory of this sibling pair suggests that initiating therapy within the first year of life could represent a critical window of opportunity to prevent severe cervical myelopathy.

## Author Contributions

J.M.K. conceived and designed the study. S.C. and J.M.K. analyzed and interpreted the data and drafted the manuscript. J.M.K., H.K., and T.‐J.C. critically revised the manuscript for important intellectual content and supervised the study. All authors read and approved the final manuscript.

## Funding

The authors have nothing to report.

## Ethics Statement

Ethical approval was waived by the Institutional Review Board of Seoul National University Hospital owing to the retrospective design and case report format. Written informed consent for genetic analysis and publication was obtained from the family because the patients were underage.

## Consent

Informed consent for preparation of this case report was obtained from the family.

## Conflicts of Interest

The authors declare no conflicts of interest.

## Data Availability

The data that support the findings of this study are available from the corresponding author upon reasonable request.
